# Laser Ablation Electrospray Ionization Time-of-Flight Mass Spectrometry for Direct Analysis of Biological Tissue

**DOI:** 10.1155/2019/1417035

**Published:** 2019-10-29

**Authors:** Wenzhao Zhou, Yan Hong, Chaoqun Huang, Chengyin Shen, Yannan Chu

**Affiliations:** ^1^Anhui Province Key Laboratory of Medical Physics and Technology, Center of Medical Physics and Technology, Hefei Institutes of Physical Science, Chinese Academy of Sciences, Hefei, Anhui 230031, China; ^2^School of Electrical and Information Engineering, Anhui University of Science and Technology, Huainan, Anhui 232001, China

## Abstract

Direct analysis and identification of biological tissue is significant for clinical applications. In this study, porcine liver and kidney have been analyzed using laser ablation electrospray ionization time-of-flight mass spectrometry (LAESI-TOFMS). This method showed good reproducibility for the same types of tissue and is capable of distinguishing different tissue species. The margin assessment was also performed using porcine renal tissue, and the response time was less than 6 s. Furthermore, human hepatocarcinoma tissue and normal tissue were identified using this method. Our results indicate that LAESI-TOFMS is a feasible approach for direct identification of tumor tissue and potential for assessment of the resection margin.

## 1. Introduction

Biological tissue contains various biomolecules, which provide abundant biochemical information. Rapid analysis of biological tissue is significant in clinical diagnosis, e.g., identification of abnormal tissue and investigation of biomarkers of disease [1–3]. The traditional histological method is commonly used for tissue analysis. This method is well established and has been applied to various types of tissue for assessment of tumors. It needs a relatively time-consuming procedure including biopsy and the pathologist's interpretation [4].

In the past decades, mass spectrometry (MS) has been applied for investigation of biological tissues. MS enables in-depth analysis of components like lipids and proteins in tissues. Matrix-assisted laser desorption/ionization mass spectrometry (MALDI-MS) is the most common technique for tissue analysis, which has been well established and achieved imaging of tissue [5]. However, it is an offline method that is also time consuming, and the usage of the matrix may interfere the detection of small molecular compounds in tissue. In recent years, a variety of ambient mass spectrometry (AMS) techniques have been developed and offered the possibility for direct analysis of complex samples. AMS techniques show great potential for rapid identification of biological tissue and even have been applied in intraoperative identification of tumor tissue and determination of the tumor resection margin [6]. Desorption electrospray ionization mass spectrometry (DESI-MS) is a typical AMS technique that allows rapid analysis of tissue and has been applied to intraoperative identification of tumor tissue. This method can only be used for analysis of in vitro tissues, which needs multiple biopsy procedures [4, 6]. By contrast, Rapid Evaporative Ionization Mass Spectrometry (REI-MS) can be used for direct analysis of human tissue in situ. By thermal evaporation of the tissue sample using an electrosurgical device, tumor tissue would be identified, and the margin can be assessd to assist surgical resection [7, 8]. A cavitron ultrasonic surgical aspirator (CUSA) combined with sonic spray ionization mass spectrometry also can be used for in situ analysis of the tissue sample [9]. Compared with REI-MS, this method can avoid excessive tissue damage and incidental bleeding during the tissue sample desorption. Recently, a MasSpec Pen technique was proposed by Zhang et al. for intraoperative tissue analysis. MasSpec Pen provides a more soft approach, which showed almost no observable damage on tissue [10].

Laser ablation electrospray ionization mass spectrometry (LAESI-MS) also enables in situ analysis of tissue components [11, 12]. This technique is proposed by Nemes and Vertes for imaging of metabolites in tissues [13]. It has been used for rapid analysis of metabolites in various biological tissues [12, 14]. Compared with other in situ detection techniques mentioned above, laser ablation provides a nontouch way for tissue desorption and evaporation, which is capable of ignoring the memory effect brought in from contacting with the tissue sample and avoiding possible infection through touch. In addition, the infrared laser at 2.94 *μ*m can effectively evaporate various tissues layer-by-layer [15, 16], leaving thin zones of thermally damaged tissue [17]. It would cause a relatively low damage to the in vivo tissue and is appropriate for in situ analysis.

In this work, laser ablation electrospray ionization time-of-flight mass spectrometry (LAESI-TOFMS) was used for rapid identification and margin assessment of different tissue types in porcine organs. To further exhibit its potential in clinical application, identification of hepatocarcinoma tissue and normal liver tissue was also carried out. The aim of this study is to characterize a LAESI-MS method for rapid identification of biological tissue and to show the potential of this method in tumor diagnosis and margin assessment of tumor tissue area.

## 2. Materials and Methods

### 2.1. Apparatus

**Figure 1:**
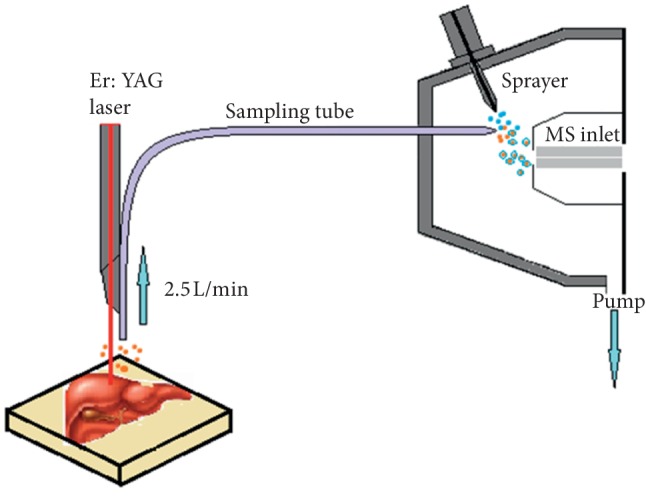
Schematic diagram of LAESI-MS for sampling.

In this study, a homemade laser ablation electrospray ion source coupled with a commercial time-of-flight mass spectrometer was used for tissue analysis. An Er:YAG laser was used for ablation, which has pulses at 2.94 *μ*m wavelength, 10 Hz repetition rate, 200 *μ*s pulse width, and spot diameter of 2 mm. As is shown in Figure 1, by simply placing the organs on the platform at about 1 cm from the tube, analytes would be ablated for sampling. A polytetrafluoroethylene (PTFE) probe was added into the electrospray ionization source chamber, and the sample vapor was driven in through room air with gas flow rate of 2.5 L/min. A pump was connected to the outlet tube for sampling. For sample ionization, the electrospray voltage (capillary voltage) was set at 4000 V, the solvent flow for spray was set at 5 *μ*L/min, and the atomizing pressure was 6 mbar.

A high-resolution mass spectrometer was used for detection (MicrOTOF-II, Bruker Daltonics Inc., Resolution >16,500 FWHM, Mass Accuracy <2 ppm), and all of the samples were analyzed in the positive-ion mode. Due to heating effect of laser ablation, some unspecific volatiles and decomposition products may appear in the low-molecular-weight range [18]. Therefore, mass spectrometry was recorded in the range of *m*/*z* 300–2000 to eliminate these interferences.

### 2.2. Samples and Reagents

Food grade porcine organs including 5 livers and 2 kidneys were purchased from local market. All samples were cut into pieces (∼30 g for one piece) and washed by pure water before test.

Tumor tissues from 3 patients with primary hepatic carcinoma were also analyzed, and these tumor tissues were sent to our laboratory for detection within 10 min after excision. Patients were recruited from the Hefei Cancer Hospital of Chinese Academy of Science. This study has been approved by the Ethical Committee of the Hefei Institutes of Physical Science, Chinese Academy of Science, and all patients gave informed consent for participation.

The solvent for electrospray was water/methanol 1 : 1 mixture containing 0.05% formic acid. Methanol and formic acid were purchased from Sigma-Aldrich, and the water was purified by using a Thermo Scientific GenPure system.

### 2.3. Data Analysis

All data were processed using the Bruker Daltonics Data analysis 4.2 software. Raw mass spectra were subtracted with air background to eliminate the interference from room air. Ion formula was established based on accurate *m*/*z* value and isotope patterns. The isotopic pattern score was presented using the mSigma value to show the deviation between the theoretical and the measured isotope pattern [18], and an mSigma value tolerance of 50 was specified for compound identification to increase the confidence of assignments. Online databases including LIPID MAPS, METLIN, and HMDB were used for compound searches, and the searching results were up to 30^th^ January, 2019. Depending on the exact ion formula, search of online databases, and reviewing of previous studies, tentative compounds of ions in mass spectra were assigned. For example, *m*/*z* 848.7683 is the most abundant ion in hepatic carcinoma tissue, and based on accurate *m*/*z* value and isotopic pattern, the ion formula can be calculated as [C_53_H_102_ NO_6_]^+^, which corresponds to NH_4_^+^ adduct ion of TAG (50 : 2) in online databases. Previous studies also detected TAG (50 : 2) in liver tissue [19]. Therefore, this ion can be established as TAG (50 : 2). It is worth noting that NH_4_^+^ adduct ions were detected in our mass spectra even without addition of ammonia. This phenomenon also has been observed in previous studies on biological tissue analysis [9, 19]. These NH_4_^+^ adduct ions may attribute to two possible reasons. First, the ammonia is desorbed from in situ tissue since ammonia commonly exits in animal tissues [20–22]. Second, the heating effect of laser ablation leads to the thermal decomposition of amino acids, which is capable of producing ammonia. Amino acids are also abundant in animal tissues [23].

Principal component analysis (PCA) was also applied to show the separation of different tissue types using Simcap 11.5 software. Ion peaks with absolute intensity over 1000 counts were used for statistics.

## 3. Result and Discussion

### 3.1. Optimizing Laser Energy

**Figure 2:**
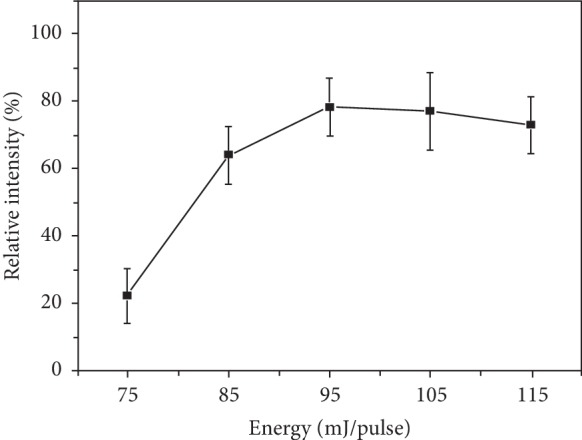
The dependence of the signal at *m*/*z* 520.5093 on laser energy.

To achieve sufficient ablation of the tissue with minimal thermal damage, laser energy was optimized for ablation using liver tissue as the test sample. The dependence of the signal at *m*/*z* 520.5093 on laser energy was investigated. The ion at *m*/*z* 520.5093 is the most abundant substance in spectra. As is shown in Figure 2, total ion intensity and the signal intensity of *m*/*z* 520.5093 increased with the laser energy increasing from 75 to 95 mJ/pulse and maintained stable afterwards. According to this result, the laser energy was set at 95 mJ/pulse as the optimal condition.

### 3.2. Analysis of Porcine Organs

**Figure 3:**
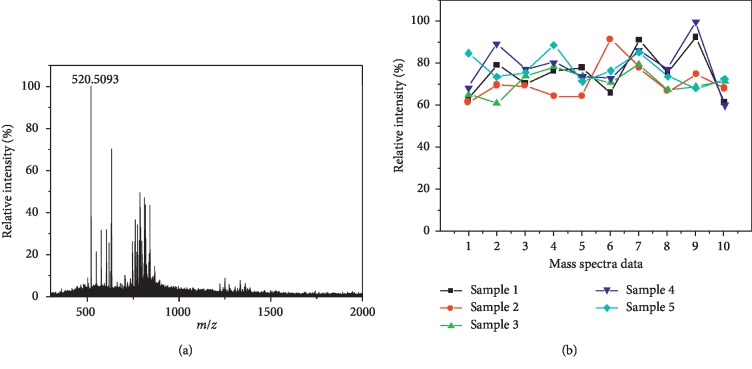
(a) Typical mass spectrum of porcine liver tissue, and (b) ion intensity of *m*/*z* 520.5093 in 50 mass spectra from 5 pieces of the liver.

Both porcine liver and kidney were analyzed in this study. To show the reproducibility of this method in tissue analysis, five pieces of liver tissue were detected, and a total of 50 mass spectra were collected with 10 from each sample. The typical spectrum of liver tissue is shown in Figure 3(a), and the signal at *m*/*z* 520.5093 has been focused. The relative standard deviation (RSD) of this component was calculated with 10 mass spectra from each piece of the liver, and the maximum RSD of 14.4% was presented. The signals of 5 liver tissue samples are also presented in Figure 3(b) and exhibited good subject-to-subject reproducibility for identical tissue type.

**Figure 4:**
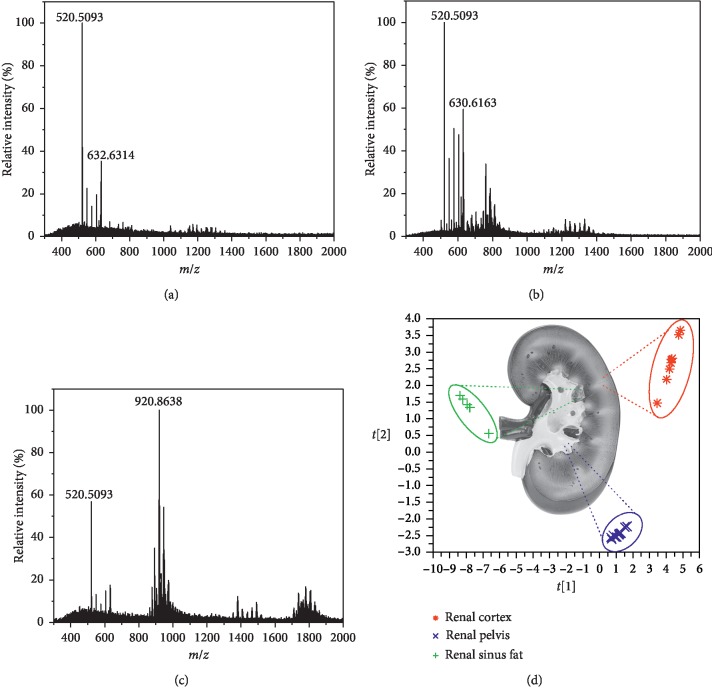
Typical mass spectra of (a) renal cortex, (b) renal pelvis, (c) renal sinus fat, and (d) the PCA plot, which explains 95.5% of the variance using three components.

To distinguish different tissues, renal tissue was also identified, and three areas with different tissue types, including renal cortex, renal pelvis, and renal sinus fat, were found to yield different characteristic spectra (Figure 4). PCA analysis was carried out with 10 mass spectra for each kind of organs, and different tissues were distinctly separated using the first two components. As can be seen in mass spectra, distinction between different tissues mainly reflected in different lipid compositions. In the renal cortex, various ceramides (Cer) and phosphatidylethanolamines (PE) were observed like Cer (34 : 1) and PE (36 : 2). Most compounds detected from renal pelvis tissue also belong to these lipid species. However, compared with the renal cortex, renal pelvis spectra presented a different ratio of *m*/*z* 630.6163 and *m*/*z* 632.6314 (Cer (42 : 2) and Cer (42 : 1)) and showed a higher intensity of phosphatidylcholines (PC) like *m*/*z* 758.5655 and 760.5832 which are assigned to PC (34 : 1) and PC (34 : 2), respectively in the mass range of 400–800. For renal sinus fat, signals appeared in the spectra were mainly triaclyglycerols (TAGs) in the mass range of *m*/*z* 800–1000. Tentative components in the porcine liver and renal tissue are listed in [Supplementary-material supplementary-material-1] in the supplementary material, which were established by the exact ion formula, searching relevant references [7, 19, 24] and online databases.

**Figure 5:**
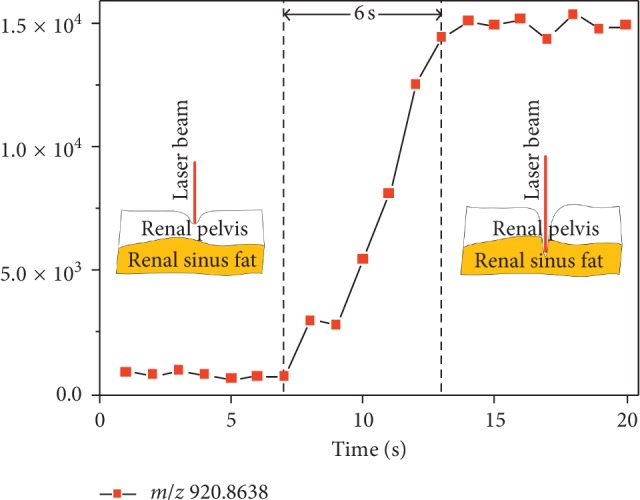
Response time for margin assessment was less than 6 s.

To further investigate the ability of this method in margin assessment, the recognition of margin between the renal pelvis and renal sinus fat was carried out. In the porcine kidney, the renal pelvis is surrounded by much renal sinus fat, and not easily distinguished. As the signal at *m*/*z* 920.8638 is the most abundant component in renal sinus fat and shows low content in the renal pelvis, it has been used to illustrate the response of spectra changes. In the beginning, by continuous ablation of the same tissue area, the signal at *m*/*z* 920.8638 remained stable in low ion intensity, which reflected the tissue of the renal pelvis. As is illustrated in Figure 5, after complete ablation of the surface of the renal pelvis, the signal intensity significantly increased within 6 s and maintained stable afterward. This result demonstrates that LAESI-MS is able to assess margin of tissue with the response time less than 6 s.

### 3.3. Identification of Hepatocarcinoma Tissue

In clinical practice, the significance of direct tissue identification lies in its potential use in tumor surgery, as it provides a rapid approach for identification of tumor tissue and assessment of tumor resection margin. Previous research studies have shown that cancer cells possess different lipid composition compared with normal cells [8, 25]. Based on the results of the porcine organ, most of the substances detected by LAESI-MS were also lipids. Therefore, this method presents great potential in identification of tumor tissue.

**Figure 6:**
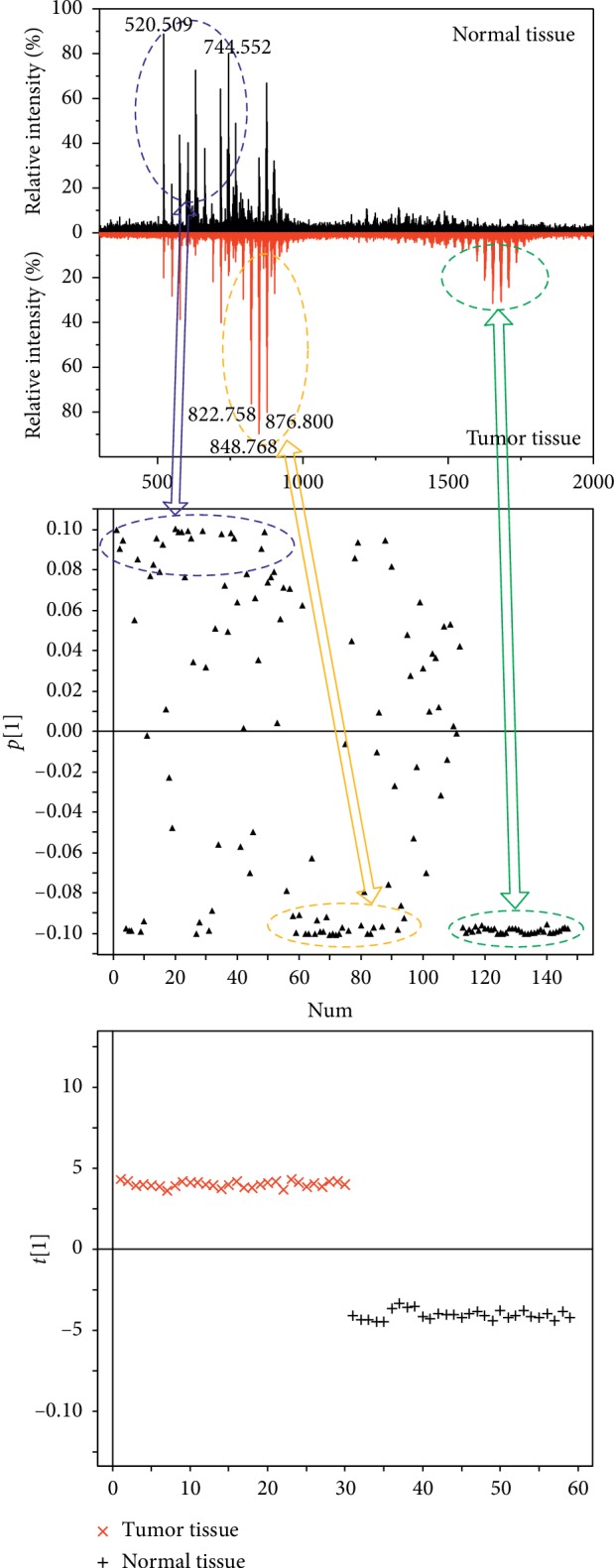
(a) Typical spectra of healthy tissue and tumor tissue, (b) the loading plot for mass spectra data with PCA, and (c) the PCA score plot explains 88.6% of the variance using one component and showed separation between healthy tissue and tumor tissue.

Analysis of hepatocarcinoma tissue and adjacent normal liver tissue was performed, and 30 mass spectra were collected from 3 cases (10 spectra from each case). Typical spectra presented significant differences in components (Figure 6(a)). These mass spectra were also analyzed using PCA to exhibit separation between hepatocarcinoma tissue and adjacent normal liver tissue (Figure 6(b)). Although requires more samples for further investigation, the preliminary result has shown potential of LAESI-MS in identification of tumor tissue. To investigate the characteristic ions that contribute to the separation, the loading plot was also presented in the Figure 6(c). As can be seen, the ion signals in the mass range of *m*/*z* 500–800, 800–1000, and 1500–1800 contribute mostly for the separation. The ions ranging from 500 to 800 were mainly ceramides, phosphatidylcholines, and phosphatidylethanolamines (e.g., Cer (34 : 1), PC (34 : 1), and PE (34 : 1)), which are dominated in the mass spectra of normal tissue.

The ions ranging from *m*/*z* 800 to 1000 belong to the triaclyglycerols, e.g., TAG (48 : 1), TAG (50 : 2), and TAG (52 : 2). These components presented high content in hepatocarcinoma tissues. According to previous studies, hepatocarcinoma cells are rich in triaclyglycerol droplets. Moreover, hepatic carcinoma also leads to triaclyglycerols increasing in plasma [26–28]. The triaclyglycerol content in hepatocytes depends in part on the rate of lipogenesis. Compared with noncancerous liver tissue, lipogenic enzymes are markedly induced in hepatocellular carcinomas and therefore lead to high content of triaclyglycerols [26]. The ions in the mass range of m/*z* 1500–1800 cannot be identified unambiguously. The formula of these ions does not match with that of any compounds in databases. These substances may be multimers rather than single molecules.

## 4. Conclusion

In this work, a LAESI-TOFMS has been used for direct analysis of biological tissue. The laser energy was optimized at 95 mJ/pulse for ablation. The mass spectra of the porcine liver tissue showed reproducibility within 14.4% (RSD), and the mass spectra of porcine renal tissue with different types presented different lipid distribution. Margin assessment using porcine renal tissue presents response time within 6 s. In addition, identification of hepatocarcinoma tissue and normal tissue was performed, and the result exhibited that tumor tissues can be distinguished from the adjacent normal parts. LAESI-MS provides a new approach for direct identification of biological tissue and may serve as a basis for future development of a clinical method to assist the tumor diagnosis.

## Data Availability

The data used to support the findings of this study are included within the article.

## Supplementary Materials

Supplementary Materials Tentative assignment of 72 ions in porcine liver tissue and kidney tissue.
