# Supplementary material for: Laser Ablation Electrospray Ionization Time-of-Flight Mass Spectrometry for Direct Analysis of Biological Tissue
Source: J Anal Methods Chem. 2019 Oct 29;2019:1417035. doi: 10.1155/2019/1417035 (PMC6854941; doi:10.1155/2019/1417035)
Supplement: Supplementary Materials — Tentative assignment of 72 ions in porcine liver tissue and kidney tissue. [file 1417035.f1.pdf]

Table S1. Tentative assignment of 72 ions in porcine liver tissue and kidney tissue

m/z	Relative error /ppm	mSigma value	Formula	Tentative assignment
520.5093	1.0	8.2	$[C_{34}H_{67}NO_3+H-H_2O]^+$	Cer(34:0)
548.5404	0.5	19.3	$[C_{36}H_{71}NO_3+H-H_2O]^+$	Cer(36:1)
576.5704	1.7	26.1	$[C_{38}H_{75}NO_3+H-H_2O]^+$	Cer(38:1)
604.6015	2.0	23.7	$[C_{40}H_{79}NO_3+H-H_2O]^+$	Cer(40:1)
618.6165	3.1	16.2	$[C_{41}H_{81}NO_3+H-H_2O]^+$	Cer(41:1)
630.6163	3.3	9.8	$[C_{42}H_{81}NO_3+H-H_2O]^+$	Cer(42:2)
632.6314	4.1	10.6	$[C_{42}H_{83}NO_3+H-H_2O]^+$	Cer(42:1)
704.6172	2.3	20.1	$[C_{44}H_{78}O_5+NH_4]^+$	DG(41:1)
744.5520	2.4	15.9	$[C_{41}H_{78}NO_8P+H]^+$	PE (36:2)
746.5701	0.9	16.5	$[C_{41}H_{80}NO_8P+H]^+$	PE (36:1)
758.5655	5.1	14.2	$[C_{42}H_{80}NO_8P+H]^+$	PC (34:2)
760.5832	2.5	17.3	$[C_{42}H_{82}NO_8P+H]^+$	PC (34:1)
768.5513	3.3	26.0	$[C_{43}H_{78}NO_8P+H]^+$	PC (35:4)
770.5704	1.3	38.5	$[C_{43}H_{80}NO_8P+H]^+$	PE (38:3)
772.5813	4.9	41.6	$[C_{43}H_{82}NO_8P+H]^+$	PE (38:2)
774.5988	2.5	36.1	$[C_{43}H_{84}NO_8P+H]^+$	PE (38:1)
782.5679	1.9	37.6	$[C_{44}H_{80}NO_8P+H]^+$	PC (36:4)
784.5827	3.1	22.9	$[C_{44}H_{82}NO_8P+H]^+$	PC (36:3)
786.5964	5.5	19.6	$[C_{44}H_{84}NO_8P+H]^+$	PC (36:2)
788.6115	6.2	34.1	$[C_{44}H_{86}NO_8P+H]^+$	PC (36:1)
790.6303	2.2	45.8	$[C_{44}H_{88}NO_8P+H]^+$	PC (36:0)
796.5824	3.4	32.7	$[C_{45}H_{82}NO_8P+H]^+$	PE (40:4)
798.5973	4.3	27.4	$[C_{45}H_{84}NO_8P+H]^+$	PE (40:3)
810.5965	5.2	42.6	$[C_{46}H_{84}NO_8P+H]^+$	PC (38:4)
812.6131	4.1	39.3	$[C_{46}H_{86}NO_8P+H]^+$	PC (38:3)
816.6444	4.0	29.0	$[C_{46}H_{90}NO_8P+H]^+$	PC (38:1)
818.6595	4.6	33.7	$[C_{46}H_{92}NO_8P+H]^+$	PC (38:0)
820.7378	1.3	41.9	$[C_{51}H_{94}O_6+NH_4]^+$	TAG (48:2)
822.7576	3.8	35.2	$[C_{51}H_{96}O_6+NH_4]^+$	TAG (48:1)
834.7522	2.8	47.2	$[C_{52}H_{96}O_6+NH_4]^+$	TAG (49:2)
836.7696	0.7	40.6	$[C_{52}H_{98}O_6+NH_4]^+$	TAG (49:1)
840.5956	6.4	24.4	$[C_{50}H_{84}NO_8P+H-H_2O]^+$	PC (42:8)
844.7395	0.7	19.8	$[C_{53}H_{94}O_6+NH_4]^+$	TAG (50:4)
846.7561	0.7	26.3	$[C_{53}H_{96}O_6+NH_4]^+$	TAG (50:3)
848.7683	1.8	21.9	$[C_{53}H_{98}O_6+NH_4]^+$	TAG (50:2)
850.7865	0.8	48.4	$[C_{53}H_{100}O_6+NH_4]^+$	TAG (50:1)
852.8024	1.1	39.0	$[C_{53}H_{102}O_6+NH_4]^+$	TAG (50:0)
864.8023	0.9	20.6	$[C_{54}H_{102}O_6+NH_4]^+$	TAG (51:1)
874.7873	1.7	13.1	$[C_{55}H_{100}O_6+NH_4]^+$	TAG (52:3)
876.8001	1.6	15.1	$[C_{55}H_{102}O_6+NH_4]^+$	TAG (52:2)
878.8144	3.1	25.4	$[C_{55}H_{104}O_6+NH_4]^+$	TAG (52:1)
888.8024	1.0	34.8	$[C_{56}H_{102}O_6+NH_4]^+$	TAG (53:3)

890.8150	2.4	28.4	$[C_{56}H_{104}O_6+NH_4]^+$	TAG (53:2)
892.8301	3.0	37.3	$[C_{56}H_{106}O_6+NH_4]^+$	TAG (53:1)
894.8453	3.5	24.9	$[C_{56}H_{108}O_6+NH_4]^+$	TAG (53:0)
902.8152	2.1	27.8	$[C_{57}H_{104}O_6+NH_4]^+$	TAG (54:3)
904.8294	3.8	23.1	$[C_{57}H_{106}O_6+NH_4]^+$	TAG (54:2)
906.8463	2.3	28.3	$[C_{57}H_{108}O_6+NH_4]^+$	TAG (54:1)
914.8142	3.2	18.6	$[C_{58}H_{104}O_6+NH_4]^+$	TAG (55:4)
916.8310	2.0	11.5	$[C_{58}H_{106}O_6+NH_4]^+$	TAG (55:3)
918.8468	1.7	9.9	$[C_{58}H_{108}O_6+NH_4]^+$	TAG (55:2)
920.8638	0.3	5.8	$[C_{58}H_{110}O_6+NH_4]^+$	TAG (55:1)
922.8755	4.6	13.9	$[C_{58}H_{112}O_6+NH_4]^+$	TAG (55:0)
928.8344	1.7	18.7	$[C_{59}H_{106}O_6+NH_4]^+$	TAG (56:4)
930.8475	1.0	22.8	$[C_{59}H_{108}O_6+NH_4]^+$	TAG (56:3)
932.8653	1.3	14.7	$[C_{59}H_{110}O_6+NH_4]^+$	TAG (56:2)
934.8831	3.6	22.2	$[C_{59}H_{112}O_6+NH_4]^+$	TAG (56:1)
940.8352	2.4	20.5	$[C_{60}H_{106}O_6+NH_4]^+$	TAG (57:5)
942.8459	2.7	26.4	$[C_{60}H_{108}O_6+NH_4]^+$	TAG (57:4)
944.8658	1.8	12.9	$[C_{60}H_{110}O_6+NH_4]^+$	TAG (57:3)
946.8827	3.2	25.0	$[C_{60}H_{112}O_6+NH_4]^+$	TAG (57:2)
948.7983	3.4	17.3	$[C_{61}H_{102}O_6+NH_4]^+$	TAG (58:8)
950.8194	2.4	29.6	$[C_{61}H_{104}O_6+NH_4]^+$	TAG (58:7)
954.8443	4.3	21.8	$[C_{61}H_{108}O_6+NH_4]^+$	TAG (58:5)
956.8666	2.6	24.4	$[C_{61}H_{110}O_6+NH_4]^+$	TAG (58:4)
958.8812	1.6	33.2	$[C_{61}H_{112}O_6+NH_4]^+$	TAG (58:3)
968.8671	3.1	29.2	$[C_{62}H_{110}O_6+NH_4]^+$	TAG (59:5)
970.8826	3.0	40.8	$[C_{62}H_{112}O_6+NH_4]^+$	TAG (59:4)
972.8980	2.7	38.9	$[C_{62}H_{114}O_6+NH_4]^+$	TAG (59:3)
974.9152	4.3	37.2	$[C_{62}H_{116}O_6+NH_4]^+$	TAG (59:2)
976.9301	3.5	32.1	$[C_{62}H_{118}O_6+NH_4]^+$	TAG (59:1)
984.8987	3.4	42.3	$[C_{63}H_{114}O_6+NH_4]^+$	TAG (60:4)
